# Daya Krishna (1924-2007)

**Published:** 2008

**Authors:** Daniel Raveh

**Affiliations:** * *Department of Philosophy, Tel-Aviv University, Jaipur, E-mail: daniraveh@gmail.com*

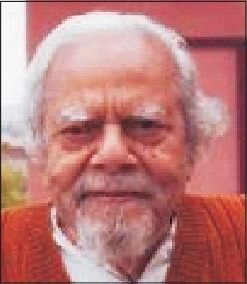



Three months have passed. I still cannot grasp the fact that he is really no longer in this world. That I will not drive to see him on my scooter, stopping on the way for a *mâlâ* of flowers, *namkeen*, lemon tart from Bake-Hut, and *pan* from the corner shop near Niro's. I used to come to see him in the evenings and always found him reading. I would sit quietly not to disturb him, until he suddenly noticed me and exclaimed: “*Kaun*”? Daniel?” We would then sit together and discuss everything under the sun: *advaita, nyâya*, Kant, beauty, mathematics, music, art, thinking itself, Pātañjala-yoga, the *Ŗgveda*, cricket. “Did you write anything?” he would ask with shining eyes or tell me of his unbelievable discoveries: about a new book or article he had read earlier in the day, about his own writing. “May I have more ice?” he would ask with a smile, pointing at his glass and then carefully, accurately, formulate another argument, encouraging me to ask a question, to disagree, to take issue with him.

## Two Great “Philosophical Battles”

Over the years Prof. Daya Krishna, or Daya*ji* as we used to call him, had been engaged in (at least) two great “philosophical battles.” The first was against what he referred to as “myths” about Indian philosophy and primarily the so-called distinction, unfortunately still prevailing in too many circles, between “Western philosophy” and “Eastern wisdom.” Daya*ji* struggled to show that Indian philosophy is no less philosophical than its Western counterpart. At the same time, he highly respected and was immensely interested in Chinese and Japanese philosophies. The reduction of Indian philosophy into a “spiritual” or *mokşa*-centered endeavour simply enraged him. In this respect, see his famous article “Three Myths about Indian Philosophy” in his Opus Magnum *Indian Philosophy–A Counter Perspective* (recently published in a revised and enlarged edition by Sri Satguru Publications, Delhi). “The interests of Western Indological studies, combined with the search for a spiritual self-identity in the face of overwhelming Western superiority in all fields of knowledge,” he wrote in his preface to this intriguing collection of articles, “seemed to have led to the creation of a certain picture of India's philosophical past, which has become fixed in the minds of successive generations both in India and abroad, through innumerable textbooks which render it almost impossible to question the picture or build a different one.”

He further writes that the second objective of his book, and of his work in Indian philosophy in general, is to “reestablish a living continuity with India's philosophical past to make it relevant to the intellectual concerns of the present.” The final objective of the book, he explains, “is to take a close look at the classical texts of the Indian philosophical traditions with unblinkered eyes.”

In other words, Daya*ji* tried-struggled-dedicated his life's work to changing reading habits and thinking patterns. Often he was frustrated to realize that his questions-queries-counter-perspective remain ignored-suppressed-“automatically rejected” by fellow scholars. This frustration finds expression in his bitingly-titled article “Shock-proof, evident -proof, argument-proof world of *sampradâyika* scholarship in Indian philosophy,” which ends with a personal statement: “I would like to add that in all intellectual matters one has to have what I have called “*nihsanga buddhi,*” which is analogous to the GÎtâ's “*nişkâma karma.*” And I may add one thing more: that for a “real” *Advaitin* it should not be difficult, for his consciousness ultimately is not attached to any specific *nâma, rűpa,* or doctrine whatsoever.”

## Last “Philosophical Battle”

Daya*ji*'s last “philosophical battle” was against postmodernism in articles such as “Narrative, Meta-Narrative, No-Narrative” (forthcoming). He believed that postmodern thinking was a childish response to the rejection of Western “modernist” attempts to “change the world” in terms of democracy, secularism, liberalism, and “reason and values.” The fact that these attempts were rejected by non-Western thinkers as colonialism and exploitation, he suggested, resulted in guilt, which led to licentiousness and total abandonment of ideals such as universality and objectivity.

Daya*ji* lived (and it is so difficult to write about him in the past tense) in a world which interlaced the philosophical and the personal. When he was ill he tried to deeply understand the notion of illness. To his friend R.C. Shah he wrote after severe illness:

I hope your worry about my illness is over, as “illness” seems to be a “natural” condition of life at all its stages and ages… The stranger problem is that there are not only “diseases” of life or what may be called the “body”, but also of “mind,” “reason,” “intellect,” and even of the “spirit.” Mental illnesses are fairly well known, but not so the diseases which “reason” itself is prone to. As for the “spiritual illnesses” one hardly hears about them. But what about imagination? Does it play an important role in diseases at all these levels? and, if so, what is the “cure” for those diseases which are rooted in imagination and, at another level, in consciousness itself? If consciousness is a “disease” that has affected life, then could not “life” be considered a “disease” that has affected matter? Matter itself does not seem to have any diseases, though there is such a thing as “rusting” or “radioactive decay” which, in any case, have not been seen as such. At one time, the story of the Black Hole seemed to suggest something analogous to what may be called “disease” at the heart of matter, but as Stephen Hawking has gone back on his earlier theory, as recently reported in the newspapers, we can hardly see it as such. You are a writer; why not write on this? Is literature a disease of language just as, according to Wittgenstein, philosophy is a disease which language suffers from when it takes a holiday from its daily use? The present stage of media and the art and even of philosophy is an evidence for this in a preeminent manner. Something has happened to man's consciousness and he is finding it difficult to get rid of the diseases of imaginations which have taken hold of it and make him do that which he could not have imagined himself doing earlier. That which was seen as the centre of creativity once, now appears just the opposite and man seems to be imprisoned in the creations of his imagination from which he can not get free…

Hence his focus was not on me and myself, about *my* illness, but rather about understanding, always about understanding.

## A Prolific Writer

Daya Krishna was a prolific writer. He published over two hundred articles on various topics: philosophy, sociology, historiography, economy, and polity, even literature. He edited the *Journal of the Indian Council of Philosophical Research* for two decades and not merely published an article-per-volume but also wrote the “Focus” and “Agenda for Research” columns, in which he tried to forge new philosophical paths and encourage fresh thinking. The notion of *samvâda*, “live dialogue” or “open debate,” was an essential ingredient of Daya*ji*'s philosophical work. In this respect, take a close look at three of his books: *Samvâda–a Dialogue between two Philosophical Traditions* (Ed. with M.P. Rege, R.C. Dwivedi, and Mukund Lath, New Delhi: Indian Council of Philosophical Research in association with Motilal Banarsidass, 1991), *Bhakti: A Contemporary Discussion–Philosophical Explorations in the Indian Bhakti Tradition* (Ed. with Mukund Lath and Francine E. Krishna, New-Delhi: Indian Council of Philosophical Research, 2000) and *Discussion and Debate in Indian Philosophy: Vedânta, MÎmâmsâ and Nyâya* (Ed., New Delhi: Indian Council of Philosophical Research, 2004). In these books and elsewhere, he was trying (with others) to establish a dialogue between traditional Pundits, thinking and writing in Sanskrit, and scholars who “do” Indian philosophy in English. Daya*ji* was furious at scholars who saw the Sanskritists as mere “informants”, and saw the necessity of a real dialogue between equals to create-achieve something new-adventurous-comprehensive in Indian philosophy. At the other end, the Pundits had to be convinced that an encounter with “Western thinking,” even by eminent Indian scholars, was significant-profound-meaningful enough for them to take part in. And of course there was the question of translation, not merely language-wise.

## The Last Two Years

In the last two years or so, Daya*ji* worked on what he referred to as *The Jaipur edition of the Ŗgveda*, an impressive project, recently completed, which again echoes the dialogue with the Sanskritists; in this case, not merely contemporary Pundits (to whom he was eager to present his work and hear their response) but also *Ŗşis* and *Ŗşikās* of the past. It was indeed his grand finale: he has taken the *âgveda*, the most revered text of the Indian tradition and put it (literally) under his magnifying glass to figure out what hides between the lines of this profound text. After a close reading, he decided to reedit the text according to the *âşis* and the *âşi*-families. He therefore purchased big scissors and started to (again literally) cut-and-paste this voluminous text. He added a longish introduction to justify the whole exercise, solve old riddles such as that of the seven *âşis* and to underscore fundamental issues such as the relationship between *âşi* and *Devata*. He has come up with a collage which is not merely original, insightful, and often fascinating, but also far-reaching, as far as its consequences are concerned, with regard to Tradition Texts in general. Between the Western monotheistic ear on the one hand and the Indian Advaitic ear on the other, we have become habituated to a certain “conventional,” “orthodox” picture of the so-called canonical texts and hence are “deaf” and “blind” with regard to counter voices-versions-readings which have the capacity of shedding new light on what we have become used to calling “*Veda,*” “*Upanişads,*” “*Bible,*” “*Koran,*” etc. Therefore the *The Jaipur edition of the âgveda* is not merely a case-study but also an “open invitation” or a prolegomena to any future experiment in rereading as rewriting in the Indian tradition and beyond.

Daya*ji*'s philosophical work deserves close attention. The craft of “questionology” and the spirit of dialogue to which he dedicated his life should, and hopefully will, be taken forward.

## About the Author

Daniel Raveh PhD is a lecturer, Department of Philosophy, Tel-Aviv University. He studied under and worked with Prof. Daya Krishna for most of the the last decade.

